# Representative scale-invariant characteristics of male and female brains in magnetic resonance images

**DOI:** 10.1016/j.ynirp.2025.100267

**Published:** 2025-06-11

**Authors:** Matthew Toews, Talía Vázquez Romaguera, William Wells, Nikos Makris

**Affiliations:** a Ecole de Technologie Superieure, Montreal, Quebec, Canada; b Harvard Medical School, Boston, MA, USA; c Massachusetts Institute of Technology, Boston, MA, USA

**Keywords:** Human brain, Anatomical MRI, Sex differences

## Abstract

This paper investigates the link between sex and the human brain from anatomical MRI data, where a primary confound is the size difference between male and female groups. Anatomy is characterized by the 3D scale-invariant feature transform (SIFT), where features are salient image regions that are automatically identified and normalized according local size or scale. Experiments use T1-w MRI data of 422 healthy unrelated subjects from the Human Connectome Project (HCP) dataset (211 males, 211 females, 22–36 years of age). We found that brain sex may be predicted via image-to-image feature matching with 91.9% accuracy, that classification is driven largely by weakly-informative features distributed throughout the brain and shared by unique subsets of subjects, and that a pair of features identified in the right and left thalamic regions of 97% of subjects predicts sex with 74% accuracy. Misclassified subjects exhibit features typical of the opposite sex in one or both hemispheres.

## 1 Introduction

Biological sex is a critical variable in neuroscience impacting both normal gender-related behaviour (Hines, 2020) and mental health disorders (McCarthy, 2016) including the variable prevalence of autism spectrum disorder (Ferri et al., 2018) and schizophrenia (Abel et al., 2010). Do male and female brains differ in terms of anatomy, and if so how? On the one hand, relatively subtle sex-related neuroanatomical differences are well-known, for example the interthalamic adhesion (ITA), aka massa intermedia, tends to be larger and more prevalent in women than men (de Macedo, 1889, Allen and Gorski, 1991, Wong et al., 2021). However, size is a major confound, as the male anatomy is on average larger than the female, and a recent survey suggested that few meaningful differences persist between male and female brains after correcting for size differences (Eliot et al., 2021). Deep network methods are able to discriminate between male and female brain MRIs with high accuracy even following normalization for size differences (Ebel et al., 2023, Gao et al., 2019), however these may be difficult to interpret due to the black box nature of deep network processing (Rudin, 2019) and it is unclear which structures might be most representative of male and female brains (Eliot et al., 2021, Ebel et al., 2023, Gao et al., 2019). While overall classification accuracy is of interest, our the primary goal of this study is to understand the neuroanatomical features driving sex classification independently from known sex-related size differences (Eliot et al., 2021).

This paper reports on representative characteristics of male and female human brains identified using the 3D scale-invariant feature transform (SIFT) method (Toews and Wells, 2013, Rister et al., 2017). In contrast to typical analyses focusing on standard neuroanatomical structures of interest, the SIFT method is based on generic salient regions of interest that may not be shared by all subjects but that when present may be detected in a manner invariant to the image scale or resolution. The classic 2D SIFT method is widely used in the field of computer vision to identify point-to-point matches between different photographs of the same scene or object despite differences in size or image resolution (Lowe, 2004). Similarly, in our previous work we have found the 3D SIFT method to be effective in 3D brain MRI tasks, e.g. identifying pairs of family members (Chauvin et al., 2021) and repeated subject scans in large public datasets (Chauvin et al., 2020). High sex classification accuracy (AUC = 95) was reported using 3D SIFT (Chauvin et al., 2021) with data from Human Connectome Project (HCP) dataset (Van Essen et al., 2013). These findings led us to hypothesize that sex classification was being driven by informative local features throughout the brain, and classification accuracy values were reproduced here to validate the current independent study into the nature of these features.

The remainder of this paper investigates sex classification of T1-w human brain MRIs using 3D SIFT features, where classification is driven by image-to-image feature matches identify the salient anatomical regions of interest. We note that in the context of this work, sex refers to self-reported binary categorization as either male or female, as provided by subjects in the HCP dataset (Van Essen et al., 2013). Gender refers to socially constructed roles, behaviours and identities related to women, men and gender-diverse individuals, and is not studied here.

## 2 Methods

We investigate the sex classification of anatomical brain MRI data based on nearest-neighbour SIFT feature matching, as described in Chauvin et al. (2021), including the overall classification accuracy and the interpretation of matches driving classification. Classification is based on a continuous sex prediction score estimated from nearest-neighbour feature matches between a subject and a set of labelled training subjects stored in memory (Cover and Hart, 1967). Intuitively, feature matches indicate distinctive anatomical similarities between subjects, and the similarity between a subject and male or female groups is quantified by their proportions of shared feature matches using the Jaccard index.

Our analysis represents each image as a set of salient keypoints, which are identified via the 3D scale-invariant feature transform (SIFT) method (Toews and Wells, 2013, Rister et al., 2017). Each SIFT feature is an image region detected at a location (x,y,z) and scale σ that locally maximizes the spherically symmetric Laplacian-of-Gaussian operator:

(1) $$\nabla^{2}I\left(x,y,z,\sigma \right)=\nabla^{2}*G\left(\sigma \right)*I\left(x,y,z\right),$$

where in Eq. (1), $\nabla^{2}$ is the Laplacian operator, G(σ) is the Gaussian kernel of standard deviation σ, I(x,y,z) is the image, and ∗ is the convolution operator. Once detected, the local image content surrounding (x,y,z) is normalized by scale σ to a standard resolution and encoded as a scale-invariant appearance descriptor from image gradient orientation information. The SIFT method was designed originally to identify corresponding keypoints in different photographs of the same scene, despite geometrical deformations due to changes in perspective and resolution (Lowe, 2004), and was used to classify sex and interpret sex differences from human face photographs acquired from arbitrary viewpoints (Toews and Arbel, 2008). The 3D SIFT-Rank method extended scale invariance to volumetric image data (Toews and Wells, 2013) and enabled a number of applications, including our own work in chest CT classification (Toews et al., 2015), ultrasound registration for image-guided neurosurgery (Machado et al., 2018), and whole-body MRI segmentation (Wachinger et al., 2018), and in other work robust mid-sagittal plane detection (Wu et al., 2014), deformable registration evaluation (Paganelli et al., 2012) and ultrasound liver image stitching (Ni et al., 2008). The strength of the SIFT method lies in the capacity to identify informative local image patterns in a manner invariant to translations, rotations and scalings of the image, with minimal image pre-processing. In studies of the human brain from anatomical MRI, it was used to identify discriminative features related to Alzheimer’s disease (Toews et al., 2010), and proved highly accurate in identifying images of family members and same-subjects from large MRI datasets (Chauvin et al., 2021, Chauvin et al., 2020) in comparison to neural networks (Puglisi et al., 2023).

With features extracted, a prediction score is derived from feature set overlap as follows. The similarity two feature sets $A=\left\{a_{i}\right\}$ and $B=\left\{a_{i}\right\}$ may be estimated via the Jaccard index or intersection-over-union J(A,B):

(2) J(A,B)=|A∩B|/(|A|+|B|−|A∩B|),

where in Eq. (2), |A∩B| is the cardinality of the set intersection A∩B of features common to both A and B. The Jaccard index lies on the range J(A,B)∈[0,1], where J(A,B)=1 indicates perfect overlap or similarity. Equivalence between a pair of features $a_{i}\in A$ and $b_{i}\in B$ is defined as according to the Euclidean distance $d\left(a_{i},b_{j}\right)=\|a_{i}-b_{j}\|$ between their appearance descriptors. The exponential function $exp^{-d^{2}\left(a_{i},b_{j}\right)}\in \left[0,1\right]$ provides a soft or non-binary measure of feature equivalence, accounting for noise and inter-subject variability, and is summed over feature matches to compute |A∩B|. The larger the number of matches between sets (A,B), the greater the Jaccard index. Our prediction score is based on the Jaccard indices J(A,Female) and J(A,Male) between a test subject feature set A and feature sets pooled from Male and Female subject MRIs. Noting that the logarithm transforms the Jaccard overlap to a distance measure −logJ(A,B)∈[0,∞], we define our prediction score as the difference of Jaccard distances $\log J\left(A,Female\right)-\log J\left(A,Male\right)\in R^{1}$ from subject feature set A to either group,

(3) Score(A)=logJ(A,Female)−logJ(A,Male),

where Score(A) generally ranges from positive or negative depending on weather A is more representative of the Male or Female group, respectively.

Experiments analysed 3D T1-weighted MRI brain scans from the human connectome project (HCP) dataset (Van Essen et al., 2013), aged 22–36 years (mean 29 years) from 434 families. A balanced set of 211 male and 211 female subjects were randomly selected from 422 different families, thereby removing potential family-related bias. Sex labels are determined based on self-reporting, HCP subjects self-reporting as female and providing complete menstrual history records (ten fields including menstrual irregularity, birth control usage, etc.) are considered female, others are considered males. Pre-processing steps such as registration or segmentation are generally not required for SIFT classification, however skull-stripped MRI data are used to restrict analysis to brain tissues.

Our analysis considers 3D SIFT features extracted from MRI volumes that are spatially registered to the MNI152 brain coordinate reference space (Mazziotta et al., 1995) according to standard HCP processing pipelines (Van Essen et al., 2013), including rigid registration (Jenkinson et al., 2002) which preserves brain size and shape, and non-linear registration which does not (Andersson et al., 2007). We compare classification from data following rigid and non-linear registration, in order to observe the effect of normalization on sex classification. We also consider classification with or without geometrical information, i.e. feature (x,y,z) centroid location and scale, where geometrically inconsistent feature matches are down-weighted in computing the Jaccard overlap and prediction score. We expect accuracy to be highest in the case of rigid registration, where size and shape information may play a role, and in the case where feature geometry information is included.

## 3 Results

Results here first establish baseline classification accuracy, then interpret classification in terms of prediction score and sex-informative 3D SIFT features. Table 1 summarizes the result of classification and feature extraction. As expected, accuracy is highest in the case of rigid registration and where feature geometry information is included (0.933) and lowest in the case of non-linear registration and no geometry information (0.832). In the case of non-linear registration, accuracy is approximately 8% lower than for rigid registration and feature counts are approximately equal. This is consistent with expectation, i.e. as non-linear registration removes size and shape differences between subjects, extracted features are more homogeneous and less informative. We note that in the case of rigid registration, male images contain 16% more features per image at a given resolution, these arise from fine-scaled image information only observable in images of larger brains which are typically male. They represent spurious noisy features that do not match systematically to either sex, and removing the excess of 292−252=40 smallest-scaled features from male sets does not affect classification. We note that the SIFT-based classification results from rigidly registered data here are consistent with those previously reported (Chauvin et al., 2021), and that results from non-linearly registered data are new to this work.

**Fig. 1:**
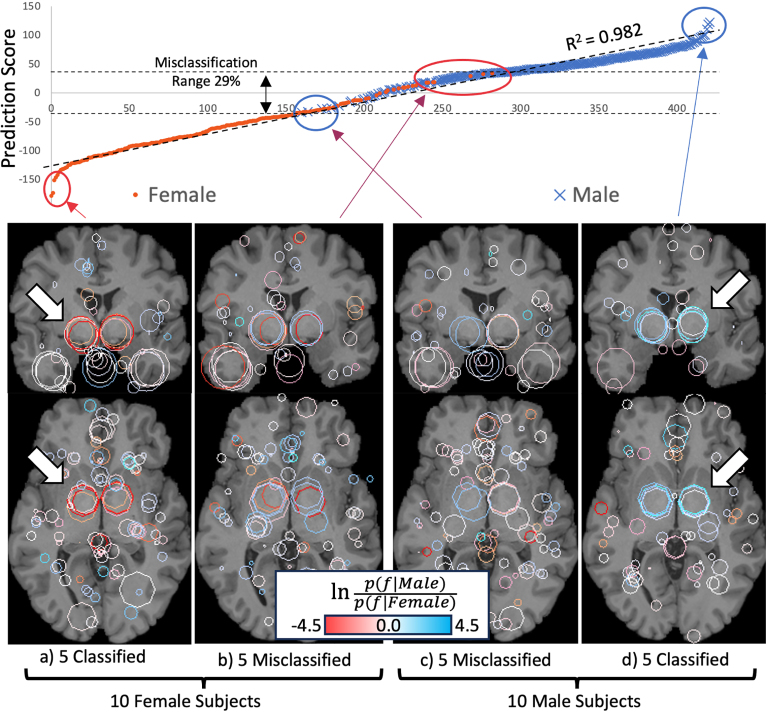
The top graph plots 422 test subjects sorted according to female (left) vs male (right) prediction score, the value of $R^{2}=0.982$ indicates a near-linear continuum. The four lower figures display sex-informative features as circles in coronal (upper) and axial (lower) slices for the 5 strongest classifications (outer pair) and misclassifications (inner pair). Feature colour indicates the log likelihood of male (blue) vs female (orange) labels associated with individual features f, white indicates equal likelihood. Two features centred approximately upon the left and right thalami (arrows) are visibly consistent with correct classification. (For interpretation of the references to colour in this figure legend, the reader is referred to the web version of this article.)

Subjects may be interpreted lying along a prediction score continuum as in Fig. 1, with subjects sorted from most female (left, orange) to most male (right, blue). Note the close linear fit with $R^{2}=0.98$, in comparison subjects distributed equally according to a binary-valued score would form a discontinuous step function with squared correlation of $R^{2}=0.75$. Visual examples of features (circles) associated with correct and incorrect classification are shown below. Circle colour indicates the affinity of each feature f in terms of the log likelihood ratio $\log\frac{p\left(f|Male\right)}{p\left(f|Female\right)}$, which ranges from female (orange) to neutral (white) to male (blue). In general each brain exhibits features associated with either male or female affinity, and it is their combination which determines classification (Chauvin et al., 2021). Two larger features centred approximately in the right and left thalami are visually consistent with correct classification, as indicated by arrows. These are automatically identified in the right and left thalamic regions of 97% of subjects, and thus represent informative common structure shared by most of the population.

**Table 1:** Sex classification of MRI brain features, given registration algorithm and inclusion of geometry information.

Registration	Accuracy		Features per image	
	w/o Geometry	w Geometry	Female	Male
Rigid	91.9±0.7	93.3±0.0	252±42	292±42
Non-linear	83.2±0.3	85.1±0.5	315±33	314±33

**Fig. 2:**
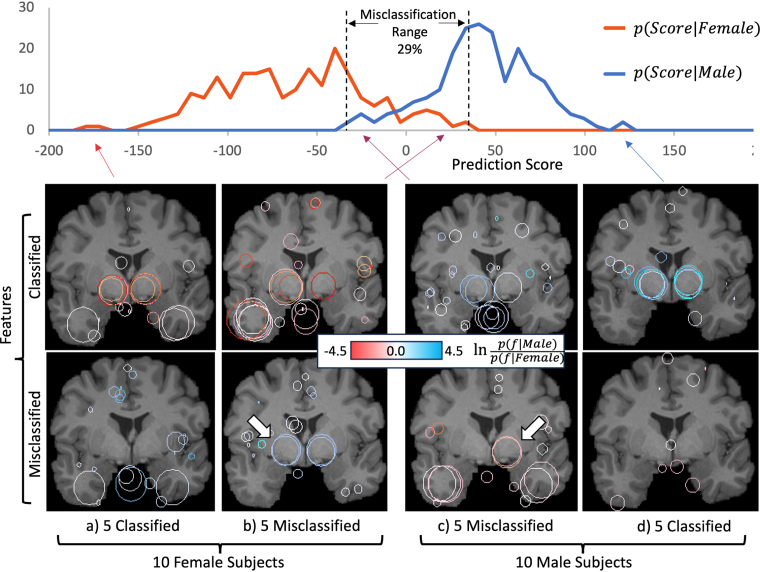
The top graph shows distributions of prediction score conditioned on female and male labels, note that misclassifications occur within an overlap range containing 29% of subjects (dashed lines). The four lower figures display sex-informative features as circles in coronal slices for the 5 strongest classifications (a) and (d), and strongest misclassifications (b) and (c). Feature colour indicates the log likelihood of male (blue) vs female (orange) labels associated with individual features, white indicates equal likelihood. The upper and lower rows display features classified or misclassified, respectively. Note that some misclassified subjects (lower row) (b) and (c) exhibit thalamic features consistent with the opposite sex. (For interpretation of the references to colour in this figure legend, the reader is referred to the web version of this article.)

Subjects may also be interpreted as clustering into prediction score distributions p(Score|Female) and p(Score|Female) conditioned on {Male,Female} labels as in Fig. 2, note that these exhibit a range of overlap associated with misclassification. Images below show examples of features associated with correctly classified subjects (a, d) and misclassified subjects (b, c). The upper and lower image rows show features that are either correctly classified or misclassified for the associated subject group. Note that misclassified subjects may exhibit thalamic features that are absent or consistent with the opposite sex, as indicated by arrows in Fig. 2 lower row. Note that the distributions appear slightly shifted to the left, e.g. the optimal classification threshold at which distributions cross is slightly lower than 0. This is due to the additional spurious male features, these increase the size of the male vs the female feature set |Male|>|Female|, which decreases the Jaccard index in Eq. (2) for the male group.

**Fig. 3:**
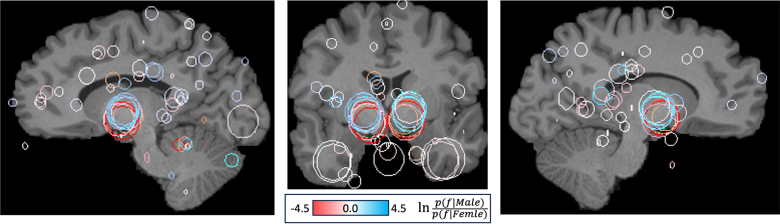
Visualizing thalamic feature variation for the five most strongly classified females and males over a representative brain MRI. Circles indicate feature location and scale, and colour indicates the log likelihood of male (blue) vs female (orange) associated with individual features. The scale difference (11.6±1.46, 11.9±1.34) between male and female features is statistically significant (p=0.0402), with a female/male size ratio of 0.968, similar to the ratio 0.970 estimated from image-to-image registration. A notable shift in z coordinate is observed due to rigid misregistration and size, this may be used to classify with 0.722 accuracy. (For interpretation of the references to colour in this figure legend, the reader is referred to the web version of this article.)

Finally, the geometry of the thalamic feature pair as identified in correctly classified subjects may be visualized together in Fig. 3 for 5 males (blue) and 5 females (orange). Note that female features (orange) are located in closer proximity, consistent with an interthalamic adhesion more frequently observed in female brains (de Macedo, 1889, Allen and Gorski, 1991, Wong et al., 2021). These two features predict sex at an accuracy of 0.69 individually and 0.74 when combined as a pair.

## 4 Discussion

Size is a primary confound in identifying meaningful anatomical differences between male and female brains in MRI data, as the male anatomy is on average larger than the female. Here we present the result of a unique feature-based analysis that recently reported high sex classification accuracy using 3D SIFT image feature matching (Chauvin et al., 2021). In 3D MRI data, SIFT features represent maximally symmetric image patterns, e.g. blob-like regions of bulging image contrast, where size or scale is determined automatically by the Laplacian-of-Gaussian detector (Toews and Wells, 2013) and used to compute image descriptors that are invariant to the local scale of the anatomy. Furthermore, classification is achieved via feature matching, which requires no explicit training procedure and allows the result to be interpreted in terms of matches between specific anatomical regions of specific individual subjects.

Baseline classification accuracy is first established from spatially normalized brain volumes from the HCP dataset (Van Essen et al., 2013), with normalization achieved via rigid and non-linear image registration. Rigid registration preserves scale and shape differences, and accuracy from scale-invariant descriptors alone is 0.919, which represents an upper limit of accuracy given no scale or location information. Accuracy rises to 0.933 if geometry information i.e. feature location and scale are included, however this may be incorporating size-related information. Classifying non-linearly registered images is a more challenging context as scale and overall shape differences are removed. Accuracy following non-linear registration is 0.832 from invariant features alone, rising to 0.851 when geometry information is included. 0.851 thus represents a lower limit of accuracy given no scale or shape information, since assuming accurate registration, geometry information serves solely to disambiguate incorrect feature matches.

Local SIFT features driving classification may be interpreted as modes of anatomy that do not occur identically in all subjects, but with a probability across a population and within groups. For example in previous work, group-informative features were identified about the hippocampi and ventricles for Alzheimer’s disease patients (Toews et al., 2010), and distributed throughout the brain and cortices for family members (Chauvin et al., 2020). Here in the case of sex classification, the most notable group informative features are a pair located symmetrically in the left and right thalamic regions. Together they predict sex with accuracy 0.74, and as they are automatically detected in 97% of subjects, they represent informative common structure shared by most subjects. This finding is consistent with prior literature, where the thalamus has been linked to sex in the human brain (de Macedo, 1889, Allen and Gorski, 1991, Wong et al., 2021) including neural networks and volume-normalized MRI data (Ebel et al., 2023, Gao et al., 2019), and also in brain MRI of other species such as mice (Spring et al., 2007). However, overall 0.919 classification appears to be driven largely by features scattered throughout the brain, a result also found in logistic regression and neural network analysis methods (Ebel et al., 2023, Gao et al., 2019). These are associated with feature matches shared by small and apparently random subsets of subjects, which are on average informative regarding sex, but which are less representative of the population as a whole.

In terms of classification accuracy, our result of 0.919 from scale-normalized feature matching appears consistent with recent literature on sex classification from volume-corrected MRIs. Logistic regression on a similar set of 399 HCP test subjects achieved 0.92 accuracy (with a model trained on an independent cohort of 3298 subjects) (Ebel et al., 2023), and SVM classifiers applied volume-normalized grey matter maps of age-matched subjects including 396 HCP subjects (1614 in total) achieved 0.925 accuracy (Wiersch et al., 2023). In terms of regions, the cerebellum and thalamus were found to be the most informative, however classification was found to be driven primarily by sex-informative features distributed throughout the brain rather than localized within individual neuroanatomical regions (Ebel et al., 2023). Our work also identified the thalamus as informative, we did not identify the cerebellum directly as it is a larger organ represented collections of smaller features, however these appear to impact overall classification similarly to other features distributed throughout the brain.

The consistency of our results with recent literature is particularly notable considering the very different prediction strategies, i.e. our method uses scale-normalized feature matching parameterized by a memory of approximately 114K features extracted from 422 subjects, vs neural network and logistic regression models with 1.3M parameters estimated 3299 volume-normalized subject images (Ebel et al., 2023). The features distributed throughout the brain and driving classification are of particular interest, observed here and in Ebel et al. (2023). In our work, these appear to be similar features shared by small and seemingly random subsets of subjects, which when identified tend to be informative regarding sex. This seems consistent with the hypothesis of brain structure development affected by multiple independent mechanisms, leading to individual brains appearing as a unique mosaics of both female and male-informative features (Joel, 2021). Feature matching offers a means of further investigating the variability of such sex-informative neuroanatomical structure that may only be observed in subsets of the population. For example, clusters of feature matches represent sparse correlations throughout the brain between subsets of subjects, and these could be characterized by alternative methods or imaging modalities to understand their role in strong overall sex prediction.

Various limitations are to be noted with regard to this work. Although features identified in the thalamic region appear informative regarding sex, we cannot further comment on their link to sex, and they may be simply the result of neurodevelopment in a larger or smaller cranial anatomy. Our investigation is limited to self-reported binary sex, and does not account for transgender or intersex individuals who do not necessarily fall into binary male or female categories. Our results are limited to the HCP cohort age range of 22–36 years, where the brain morphology is relatively stable, and does not include young or elderly subjects whose anatomy would be affected by growth and aging.

In conclusion, our findings are consistent with the interpretation that an individual level, each brain image may be viewed as mosaic of features that are generally associated with either sex (Joel, 2021). No single brain region classifies sex definitively, and classification is driven primarily by features distributed throughout the brain that may be pooled to predict sex with an accuracy of approximately 0.92. Individual subjects may be assigned a continuous sex prediction score, in our work based on the Jaccard index of feature set overlap, in other work based on regression (Ebel et al., 2023) or likelihood ratios associated with features (Phillips et al., 2019). Specific features such as the thalamic features observed here are identified in 97% of subjects and predict sex with an accuracy 0.74. Experiments with rigidly registered subjects reveal that these occur despite size differences between males and females, which are normalized out by the SIFT method here. The misclassification rate for whole-brain sex classification from feature matching lies conservatively between 9%–15%, for rigidly and non-linearly registered images, respectively. Misclassified brains are observed to exhibit features typically associated with the opposite sex.^1^

We note that this research was approved by the Research Ethics Committee at ETS, that all procedures conformed to the guidelines laid out in the most recent revision of the Declaration of Helsinki, and that all participants provided written informed consent prior to the acquisition and use of their data.

## CRediT authorship contribution statement

**Matthew Toews:** Writing – original draft, Visualization, Validation, Supervision, Software, Project administration, Methodology, Investigation, Funding acquisition, Formal analysis, Data curation, Conceptualization. **Talía Vázquez Romaguera:** Validation, Methodology. **William Wells III:** Writing – original draft, Supervision, Formal analysis, Conceptualization. **Nikos Makris:** Writing – original draft, Methodology, Formal analysis.

## Declaration of competing interest

The authors declare that they have no known competing financial interests or personal relationships that could have appeared to influence the work reported in this paper.

## Data Availability

Data will be made available on request.

## Footnotes

1 Code required to reproduce our results may be obtained at https://github.com/3dsift-rank.
